# Astragalus polysaccharide restores insulin secretion impaired by lipopolysaccharides through the protein kinase B /mammalian target of rapamycin/glucose transporter 2 pathway

**DOI:** 10.1186/s12906-023-04188-1

**Published:** 2023-10-10

**Authors:** Xiaodan Ren, Ying Dai, Mengya Shan, Jing Zheng, Zhongyi Zhang, Tao Shen

**Affiliations:** 1 School of Basic Medicine, Chengdu University of Traditional Chinese Medicine, No. 37, Shi-er-qiao Road, Jinniu District, 610075, 610075 Chengdu, Chengdu, Sichuan China; 2 Department of Integrative Medicine, Xinqiao Hospital, Army Medical University, Chongqing, China

**Keywords:** Astragalus polysaccharide, Lipopolysaccharides, Insulin secretion, Glucose transporter 2

## Abstract

**Background:**

Lipopolysaccharide (LPS)-induced dysfunction of pancreatic β-cells leads to impaired insulin (INS) secretion. Astragalus polysaccharide (APS) is a bioactive heteropolysaccharide extracted from *Astragalus membranaceus* and is a popular Chinese herbal medicine. This study aimed to elucidate the mechanisms by which APS affects INS secretion from β-cells under LPS stress.

**Methods:**

Rat insulinoma (INS-1) cells were treated with LPS at a low, medium, or high concentration of APS. Glucose-stimulated insulin secretion (GSIS) was evaluated using an enzyme-linked immunosorbent assay (ELISA). Transcriptome sequencing was used to assess genome-wide gene expression. Kyoto Encyclopedia of Genes and Genomes (KEGG) enrichment analysis was used to determine the signaling pathways affected by APS. Quantitative reverse transcription–polymerase chain reaction (qRT-PCR) was performed to evaluate the gene expression of glucose transporter 2 (*GLUT2*), glucokinase (*GCK*), pancreatic duodenal homeobox-1 (*PDX-1*), and *INS*. Western blot analysis was used to detect the protein expression of phosphorylated protein kinase B (p-Akt), total Akt (t-Akt), phosphorylated mammalian target of rapamycin (p-mTOR), total mTOR (t-mTOR), and GLUT2.

**Results:**

LPS decreased GLUT2, GCK, PDX-1, and INS expression and reduced GSIS. These LPS-induced decreases in gene expression and GSIS were restored by APS treatment. In addition, transcriptome sequencing in combination with KEGG enrichment analysis revealed changes in the INS signaling pathway following APS treatment. LPS decreased p-Akt and p-mTOR expression, which was restored by APS treatment. The restorative effects of APS on GSIS as well as on the expression of GLUT2, GCK, PDX-1, and INS were abolished by treatment with the Akt inhibitor MK2206 or the mTOR inhibitor rapamycin (RPM).

**Conclusions:**

APS restored GSIS in LPS-stimulated pancreatic β-cells by activating the Akt/mTOR/GLUT2 signaling pathway.

**Supplementary Information:**

The online version contains supplementary material available at 10.1186/s12906-023-04188-1.

## Background

The prevalence of type 2 diabetes mellitus (T2DM) is rising due to the sedentary lifestyle and unhealthy eating habits of individuals in today’s society [1]. Impaired insulin (INS) secretion due to pancreatic β-cell failure is a hallmark of T2DM pathogenesis. Drugs that increase INS secretion, such as sulfonylureas, glinides, and glucagon-like peptide-1 receptor agonists, are widely used in the management of T2DM [2]; however, these drugs often have undesirable side effects. Sulfonylureas and glinides can cause weight gain and hypoglycemia [3], while glucagon-like peptide-1 receptor agonists can cause nausea, vomiting, and diarrhea as well as increase the risk of pancreatitis [4]. Thus, there is a continuous need for novel insulinotropic drugs that can effectively stimulate INS secretion with less severe side effects.

Many components of the immune system have been implicated in pancreatic β-cell failure, including various immune cells and gut microbiota [5]. In addition, mammalian hosts with an increased intestinal permeability develop metabolic endotoxemia due to increased translocation of gut microbiota-derived lipopolysaccharides (LPS) from the intestinal lumen to the bloodstream [6]. A wealth of clinical data also has demonstrated a positive association between metabolic endotoxemia and an increased risk of T2DM [7, 8]. Moreover, microbiota-derived LPS can activate proinflammatory pathways leading to β-cell dysfunction and decreased insulin secretion [9]. Furthermore, intraportal LPS infusion in rats has been reported to cause inflammation in the liver and pancreas as well as to reduce glucose-stimulated insulin secretion (GSIS) [10]. However, the molecular mechanisms involved in LPS-induced impairment in GSIS are not fully understood.

Astragalus polysaccharide (APS) is the main active component of the Chinese herbal medicine *Astragalus membranaceus*. APS exhibits antiapoptotic [11], anti-inflammatory [12], and antitumor [13] properties. Importantly, it has been demonstrated to increase insulin secretion, reduce insulin resistance, and alleviate the symptoms of T2DM in rats [14, 15]. In *in-vitro* studies, APS has been shown to restore the proliferation and INS secretion of mouse pancreatic β-cells under high-glucose and high-free-fatty-acid stress [16], suggesting that APS may alleviate T2DM through its protective effects on β-cells.

In this study, we evaluated the effects of APS on GSIS in rat insulinoma (INS-1) cells exposed to LPS stress. Transcriptome sequencing was used in combination with Kyoto Encyclopedia of Genes and Genomes (KEGG) enrichment analysis to determine the signaling pathways affected by APS. The underlying molecular mechanisms involving the protein kinase B (Akt)/mammalian target of rapamycin (mTOR)/glucose transporter 2 (GLUT2) pathway were investigated using quantitative reverse transcription–polymerase chain reaction (qRT-PCR) and western blot analyses.

## Methods

### Chemical reagents

Sodium pyruvate, β-mercaptoethanol, and APS (> 90% pure) were purchased from Solarbio (Beijing, China). LPS, the Akt inhibitor MK2206, and the mTOR inhibitor rapamycin (RPM) were from MedChemExpress (Shanghai, China).

### Cell culture

INS-1 cells were obtained from Nanjing Kebai Biotechnology (Nanjing, China). The cells were grown in RPMI-1640 (Solarbio) containing 10% fetal bovine serum (Gibco, Thermo Fisher, USA) and 100 U/mL penicillin–streptomycin at 37 °C in a humidified CO_2_ incubator set to 5% CO_2_. The cells were passaged every 4 days. Cells at 80% confluency were used for all experiments.

### Cell counting kit-8 (CCK-8) assay

INS-1 cells were seeded in 96-well plates (200 µL, 8000 cells/well) in complete culture medium, cultured for 24 h, and then treated for 24 h with 0 or 10–5000 µg/mL APS. The cell viability was evaluated using the CCK-8 assay (Solarbio), according to the manufacturer’s instructions. The following formula was used to calculate the cell viability: Viability (%)=[OD_APS_–OD_blank_]/[OD_control_–OD_blank_]×100, where OD indicates the optical density at 450 nm.

### Apoptosis assay

INS-1 cells were seeded in 6-well plates at a density of 3.5 × 10^5^ cells/well, cultured for 24 h, and then treated for 24 h with 10 µg/mL LPS (LPS) alone or 10 µg/mL LPS + 40, 80, or 160 µg/mL APS (LPS + APS-L, APS-M, or APS-H). Untreated cells were included as a control (CON). The concentration of LPS was chosen based on previous reports [17, 18] and the results from our preliminary experiments. After the treatment was completed, the cells were subjected to flow cytometric analysis for determining apoptosis using a PE Annexin V Apoptosis Detection Kit (Beyotime, Shanghai, China).

### INS secretion assay

INS-1 cells were seeded in 24-well plates at a density of 5 × 10^4^ cells/well, incubated for 24 h, and then treated for 24 h with LPS alone or LPS + APS-L, APS-M, or APS-H as above. To test the effects of inhibitors, the cells were treated for 24 h with LPS + APS-H + 1 µM MK2206 (LPS + APS-H + MK2206) or 5 µM RPM (LPS + APS-H + RPM). Untreated cells were included as a control (CON). After the treatment was completed, the cells were starved in glucose-free Krebs–Ringer buffer containing 1% bovine serum albumin for 2 h and subsequently stimulated with low (5.5 mM) or high (25 mM) glucose for 1 h. The INS concentrations in the supernatant were measured with an enzyme-linked immunosorbent assay (ELISA) (Solarbio), according to the manufacturer’s instructions.

### Transcriptome sequencing and KEGG enrichment analysis

INS-1 cells were seeded in 24-well plates as above, incubated for 24 h, and then treated with LPS alone or LPS + APS-H for 24 h. After the treatment was completed, the cells were sent to Nanjing Personal Gene Technology Co., Ltd. (Nanjing, China) for transcriptome sequencing and KEGG pathway enrichment analysis [19].

### qRT-PCR

INS-1 cells were seeded in 24-well plates as above, incubated for 24 h, and then treated with LPS alone, LPS + APS-L, APS-M, or APS-H for 24 h. To test the effects of the inhibitors, the cells were treated for 24 h with LPS + APS-H + 1 µM MK2206 (LPS + APS-H + MK2206) or 5 µM RPM (LPS + APS-H + RPM). Untreated cells were included as a control (CON). After the treatment was completed, the total RNA was extracted with Trizol reagent (Beyotime) and reversely transcribed into cDNA using a cDNA synthesis kit (TaKaRa, Japan). qRT-PCR was performed with SYBR-green PCR Master Mix on a Fast Real-time PCR 7500 System (Applied Biosystems, USA). Data were normalized to glyceraldehyde 3-phosphate dehydrogenase (*GAPDH*). The PCR primers used for RT-PCR were as follows: *INS*, forward, CCTGCCCAGGCTTTTGTC, reverse, TTGCGGGTCCTCCACTTC; tumor necrosis factor alpha (*TNF-α*), forward, CTCTTCTCATTCCCGCTCGT, reverse, GGGAGCCCATTTGGGAACTT; interleukin-1β (*IL-1β*), forward, CCTCGTCCTAAGTCACTCGC, reverse, GCAGAGTCTTTTTGACCCTCCT; pancreatic duodenal homeobox-1 (*PDX-1*), forward, AACGCTGGAACAGGGAAG, reverse, CACGGGAAAGGGAGATGA; glucokinase (*GCK*), forward, CGGTGGGAAGTATATGGGCG, reverse, TGTGGATCTGCTTTCGGTCC; *GLUT2*, forward, TTAGCAACTGGGTCTGCAAT, reverse, GGTGTAGTCCTACACTCATG; *GAPDH*, forward, CCTTCATTGACCTCAACTACATGG, reverse, ATGGCATGGACTGTGGTCATGAG.

### Western blot analysis

INS-1 cells were seeded in 6-well plates as above, incubated for 24 h, and then treated for 24 h with LPS alone or LPS + APS-L, APS-M, or APS-H. To test the effects of the inhibitors, the cells were treated for 24 h with LPS + APS-H + 1 µM MK2206 (LPS + APS-H + MK2206) or 5 µM RPM (LPS + APS-H + RPM). Untreated cells were included as a control (CON). After the treatment was completed, the cells were lysed in radioimmunoprecipitation assay lysis buffer (Beyotime) containing phosphatase and protease inhibitors (Beyotime) on ice for 20 min. The lysates were centrifuged at 12,000 *g* and 4 °C for 20 min. The protein concentrations in the supernatants were determined using the bicinchoninic acid method. The proteins were separated by sodium dodecyl sulfate–polyacrylamide gel electrophoresis (SDS-PAGE) and transferred to polyvinylidene difluoride membranes (Millipore, USA). After blocking in a blocking solution (Wellget, Shanghai, China) for 30 min, the membranes were incubated with a primary antibody (1:1000) at 4 °C overnight, and then a secondary antibody at room temperature for 2 h. The primary antibodies against phosphorylated Akt (p-Akt) (1:1000, #5012), total Akt (t-Akt) (1:1000, #4691), phosphorylated mTOR (p-mTOR) (1:1000, #5536), and total mTOR (t-mTOR) (1:1000, #2983) were from Cell Signaling Technology (USA). The primary antibody against GLUT2 (1:200, #sc-518,022) was from Santa Cruz Biotechnology (USA). The primary antibody against GAPDH (1:50,000, #60004-1-Ig) was from Proteintech (Wuhan, China). The secondary antibody (1:1000, #ab7097) was from Abcam (UK). After washing three times with tris-buffered saline containing Tween 20 (Solarbio), the protein bands were detected with enhanced chemiluminescence reagent (Bio-Rad, USA) on an ImageQuant LAS 4000 imager (GE Healthcare Life Sciences, USA). Image J software was used to quantify the protein bands.

### Statistical analysis

All results are presented as the mean ± standard deviation. Data were analyzed with GraphPad Prism-5 software and compared using the Student’s *t*-test or analysis of variance followed by the Tukey’s post-hoc test. Differences with a *P*-value less than 0.05 were deemed statistically significant.

## Results

### Effects of APS on INS-1 cell viability

**Fig. 1:**
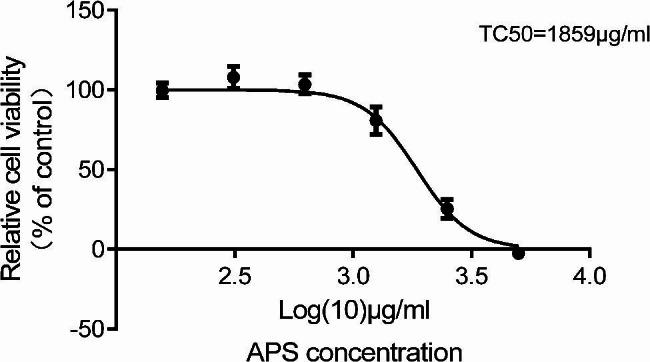
The effects of APS on INS-1 cell viability. The TC50 was calculated to be 1859 µg/mL. TC50: concentration inducing 50% cell mortality

The effects of APS at concentrations of 10–5000 µg/mL on INS-1 cell viability were evaluated by the CCK-8 assay. After a 24-h treatment, APS showed no significant cytotoxicity at concentrations up to 800 µg/mL. At higher concentrations, APS significantly reduced the cell viability, with a concentration inducing 50% cell mortality (TC50) value of 1859 µg/mL (Fig. 1). Considering the experimental conditions used in previous literature reports [20] and our pre-experimental results, APS was tested at concentrations of 40 µg/mL (low), 80 µg/mL (medium), and 160 µg/mL (high) in subsequent experiments.

### APS restored GSIS of INS-1 cells impaired by LPS

**Fig. 2:**
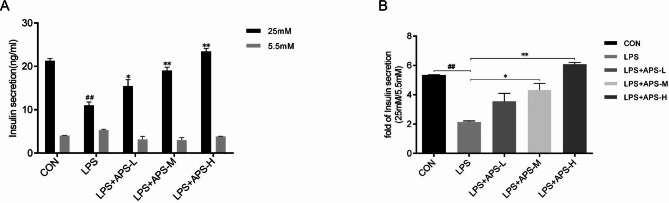
APS restored GSIS impaired by LPS. INS-1 cells were pretreated as indicated for 24 h, starved for 2 h, and subsequently incubated in low-glucose (5.5 mM, LG) or high-glucose (25 mM, HG) medium for 1 h. CON: blank control; LPS: 10 µg/mL LPS; LPS + APS-L: LPS + low-dose APS (40 µg/mL); LPS + APS-M: LPS + medium-dose APS (80 µg/mL); LPS + APS-H: LPS + high-dose APS (160 µg/mL). **(A)** INS concentrations in the culture supernatant by ELISA. *n* = 3;^##^*P* < 0.01 vs. CON; *P < 0.05, **P < 0.01 vs. LPS. **(B)** The ratio of the INS concentration in HG medium to that in LG medium. *n* = 3, *P < 0.05, **^, ##^*P* < 0.01

To evaluate GSIS, INS-1 cells were starved for 2 h and subsequently incubated in high-glucose (25 mM) or low-glucose (5.5 mM) medium for 1 h. The INS concentrations in the culture supernatant were determined by ELISA. It was found that a 24 h prestimulation with 10 µg/mL LPS significantly reduced GSIS (*P* < 0.01, Fig. 2A). Importantly, preincubation with a low, medium, or high concentration of APS for 24 h dose-dependently restored GSIS in cells exposed to LPS stress, especially in the high-APS-concentration group (*P* < 0.01, Fig. 2A). Notably, APS showed no effects on basal insulin secretion (BIS, insulin secretion under low glucose) (Fig. 2A). This was not a surprise given that GSIS and BIS are regulated by distinct cellular and molecular mechanisms [21, 22]. The GSIS/BIS ratio indicates the ability of β cells to respond to nutrient stimulation, a fundamental β-cell function. As shown in Fig. 2B, APS dose dependently restored the GSIS/BIS ratio impaired by LPS, indicating that APS protected against LPS-induced β-cell dysfunction.

### Transcriptome sequencing and KEGG pathway enrichment analysis

**Fig. 3:**
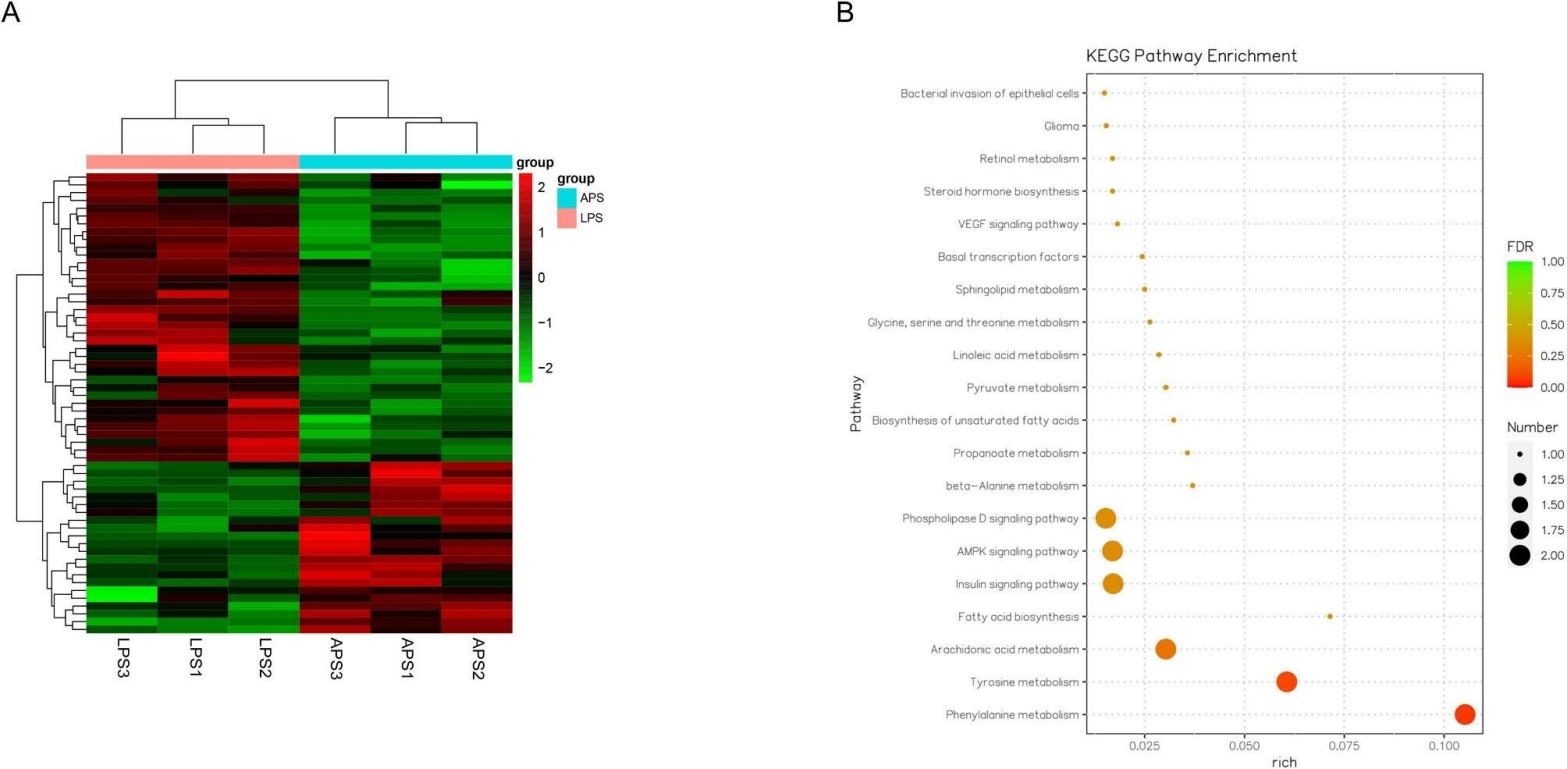
Transcriptome sequencing and KEGG pathway enrichment analysis. INS-1 cells were treated with LPS or LPS + APS-H for 24 h. **(A)** The transcriptome sequencing heatmap of data from three independent experiments. APS represents LPS + APS-H. **(B)** KEGG pathway enrichment analysis

Flow cytometric analysis showed that neither LPS (10 µg/mL) nor APS (low, medium, or high concentration) had any significant effects on apoptosis (Sup. Figure 1). Considering that LPS can induce inflammatory responses in many cell types, qRT-PCR was used to evaluate the inflammatory factors TNF-α and IL-1β. Indeed, a 24-h stimulation with 10 µg/mL LPS significantly induced these inflammatory factors (*P* < 0.05, Sup. Figure 2). However, APS further upregulated *TNF-α* and *IL-1β* (*P* < 0.01, Sup. Figure 2). Thus, the restorative effects of APS on GSIS could not be explained by its immunoregulatory properties. To fully uncover the molecular mechanisms of APS, transcriptome sequencing was used to identify genes differentially expressed between cells treated with LPS (LPS) alone and those treated with LPS in combination with APS-H. The resulting heatmap is presented in Fig. 3A (APS represents LPS + APS-H). The genes that showed differential expression between the LPS and APS groups of cells were subjected to KEGG pathway enrichment analysis. Among the pathways most profoundly affected by APS (Fig. 3B), the INS signaling pathway was selected for further investigation. *GLUT2*, *GCK*, and *PDX-1* are genes in the INS signaling pathway that regulate GSIS. The transcriptome sequencing data revealed significantly greater *GLUT2*, *GCK*, *PDX-1*, and *INS* levels in the APS cells compared to the LPS cells (Fig. 3A). Thus, these genes and proteins were further investigated by qRT-PCR and western blot analyses, respectively, in subsequent experiments.

### APS restored GLUT2, GCK, PDX-1, and INS downregulated by LPS

**Fig. 4:**
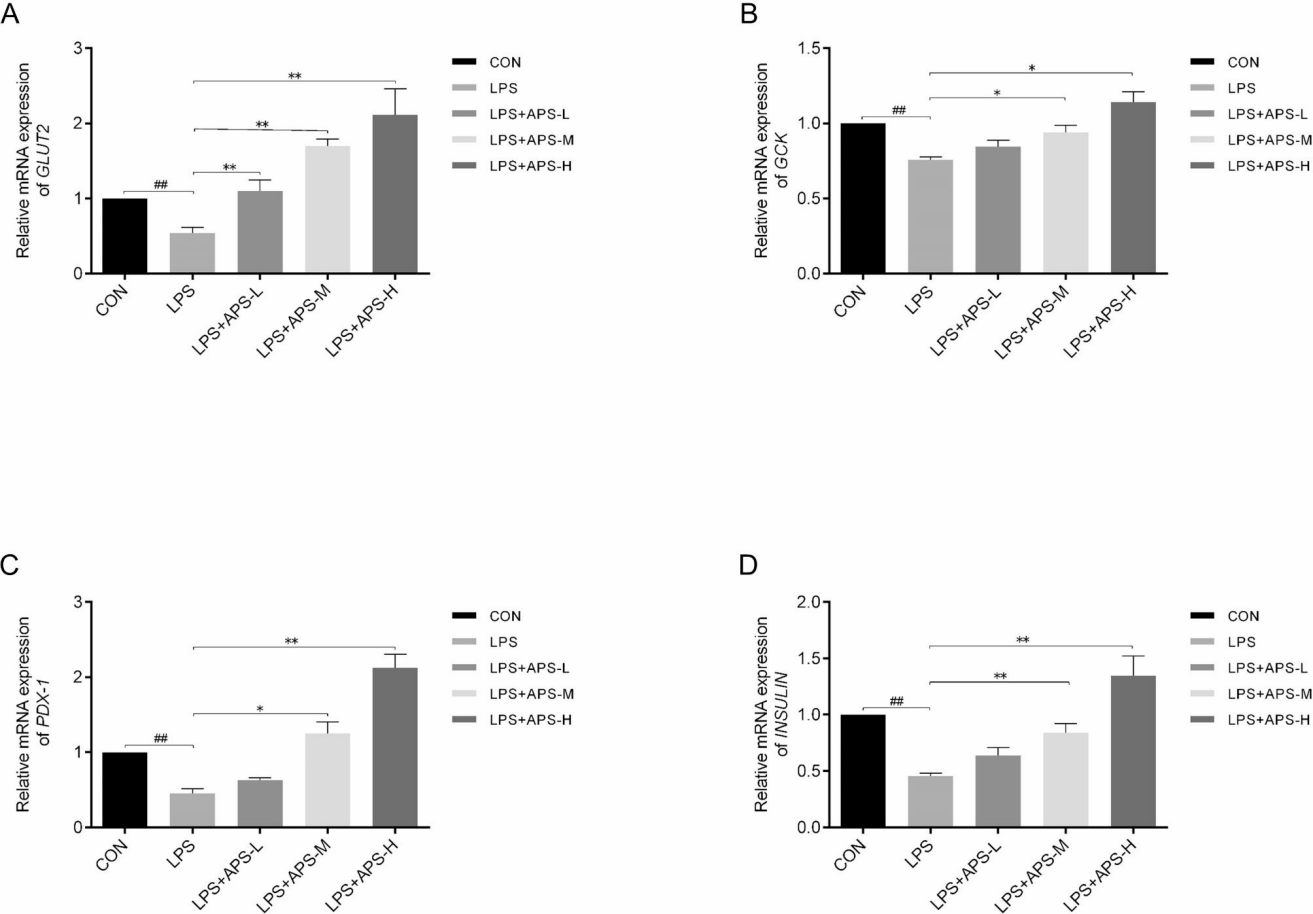
APS restored *GLUT2*, *GCK*, *PDX-1*, and *INS*downregulated by LPS. INS-1 cells were treated as indicated for 24 h. The mRNA levels of *GLUT2* **(A)**, *GCK* **(B)**, *PDX-1* **(C)**, and *INS* **(D)** were determined by qRT-PCR. *n* = 3, *P < 0.05, **^, ##^*P* < 0.01

The qRT-PCR results revealed that a 24-h stimulation with 10 µg/mL LPS significantly downregulated *GLUT2*, *GCK*, *PDX-1*, and *INS*, while treatment with a low, medium, or high concentration of APS dose-dependently restored the expression of these genes in cells exposed to LPS stress (*P* < 0.05, Fig. 4A–D). These findings confirmed the transcriptome sequencing results and suggested that APS restored GSIS through the upregulation of *GLUT2*, *GCK*, *PDX-1*, and *INS*. Given that GLUT2 is the predominant glucose transporter in pancreatic β-cells and a master regulator of GSIS [23, 24], GLUT2 was selected as a representative GSIS-related gene for further investigation.

### APS restored GLUT2 downregulated by LPS via the Akt/mTOR pathway

**Fig. 5:**
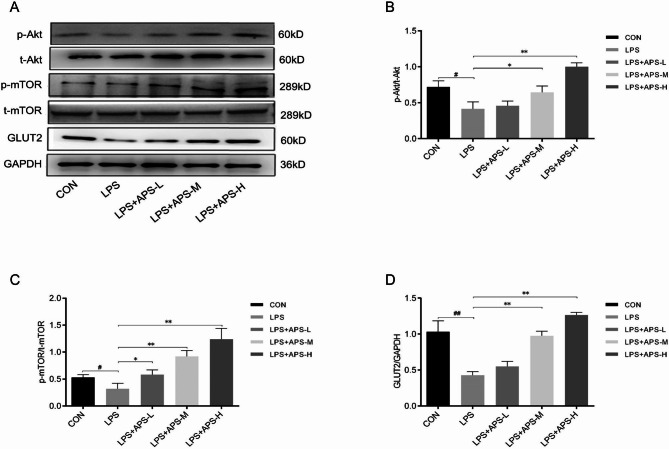
APS restored GLUT2 downregulated by LPS via the Akt/mTOR pathway. INS-1 cells were treated as indicated for 24 h. The levels of the indicated proteins were determined by western blot analysis. **(A)** Representative gel images. **(B)** Quantified protein ratios of p-Akt/t-Akt. **(C)** Quantified protein ratios of p-mTOR/t-mTOR. **(D)** Quantified GLUT2 protein expression. *n* = 3, *^, #^*P* < 0.05, **^, ##^*P* < 0.01

Given that the expression of GLUT2 in pancreatic β-cells is controlled by the Akt/mTOR pathway [25], we speculated that this pathway may mediate the regulation of GLUT2 by LPS and APS. To test this hypothesis, western blot analysis was used to evaluate p-Akt, t-Akt, p-mTOR, t-mTOR, and GLUT2 expression in INS-1 cells. The results showed that a 24-h stimulation with 10 µg/mL LPS led to significantly reduced GLUT2 levels (*P* < 0.01, Fig. 5D) along with decreased p-Akt/t-Akt (*P* < 0.05, Fig. 5B) and p-mTOR/t-mTOR ratios (*P* < 0.05, Fig. 5C). The t-Akt and t-mTOR levels remained mostly unchanged (Fig. 5A). Treatment with a low, medium, or high concentration of APS dose-dependently restored the phospho-kinase/total kinase ratios and GLUT2 expression decreased by LPS (*P* < 0.05, Fig. 5B–D). These results suggested that APS restored GLUT2 expression in LPS-stimulated INS-1 cells through the Akt/mTOR pathway.

### APS restored GSIS impaired by LPS via the Akt/mTOR pathway

**Fig. 6:**
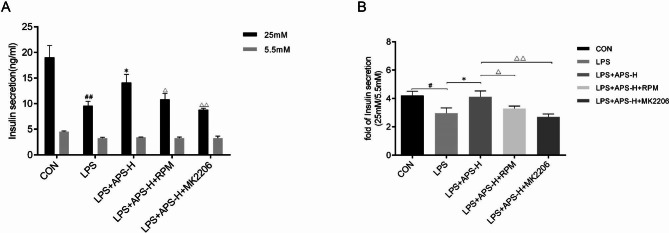
Blockade of the Akt/mTOR pathway reduced GSIS restored by APS in LPS-stimulated INS-1 cells. INS-1 cells were pretreated as indicated for 24 h, starved for 2 h, and subsequently incubated in low-glucose (5.5 mM, LG) or high-glucose (25 mM, HG) medium for 1 h. Untreated cells (CON) were included as a control. **(A)** INS concentrations in the culture supernatant by ELISA. *n* = 3; ^##^*P <* 0.01 vs. CON; *P < 0.05 vs. LPS; ^Δ^*P* < 0.05, ^ΔΔ^*P <* 0.01 vs. LPS + APS-H. **(B)** Ratio of the INS concentration in HG medium to that in LG medium. *n* = 3, ^#,^*^,Δ^*P* < 0.05, ^ΔΔ^*P <* 0.01

To investigate whether the Akt/mTOR pathway mediates the restorative effects of APS on GSIS, the Akt inhibitor MK2206 and the mTOR inhibitor RPM were used to block the pathway. Compared with INS-1 cells prestimulated with LPS + APS-H for 24 h, the cells pretreated with LPS + APS-H + 1 µM MK2206 or 5 µM RPM for 24 h showed significantly diminished GSIS and GSIS/BIS ratio (*P* < 0.05, Fig. 6A, B). Notably, MK2205 and RPM showed no significant effects on BIS (Fig. 6A). These results verified that APS restored GSIS impaired by LPS through the Akt/mTOR pathway.

### APS restored GLUT2, GCK, PDX-1, and INS expression downregulated by LPS via the Akt/mTOR pathway

**Fig. 7:**
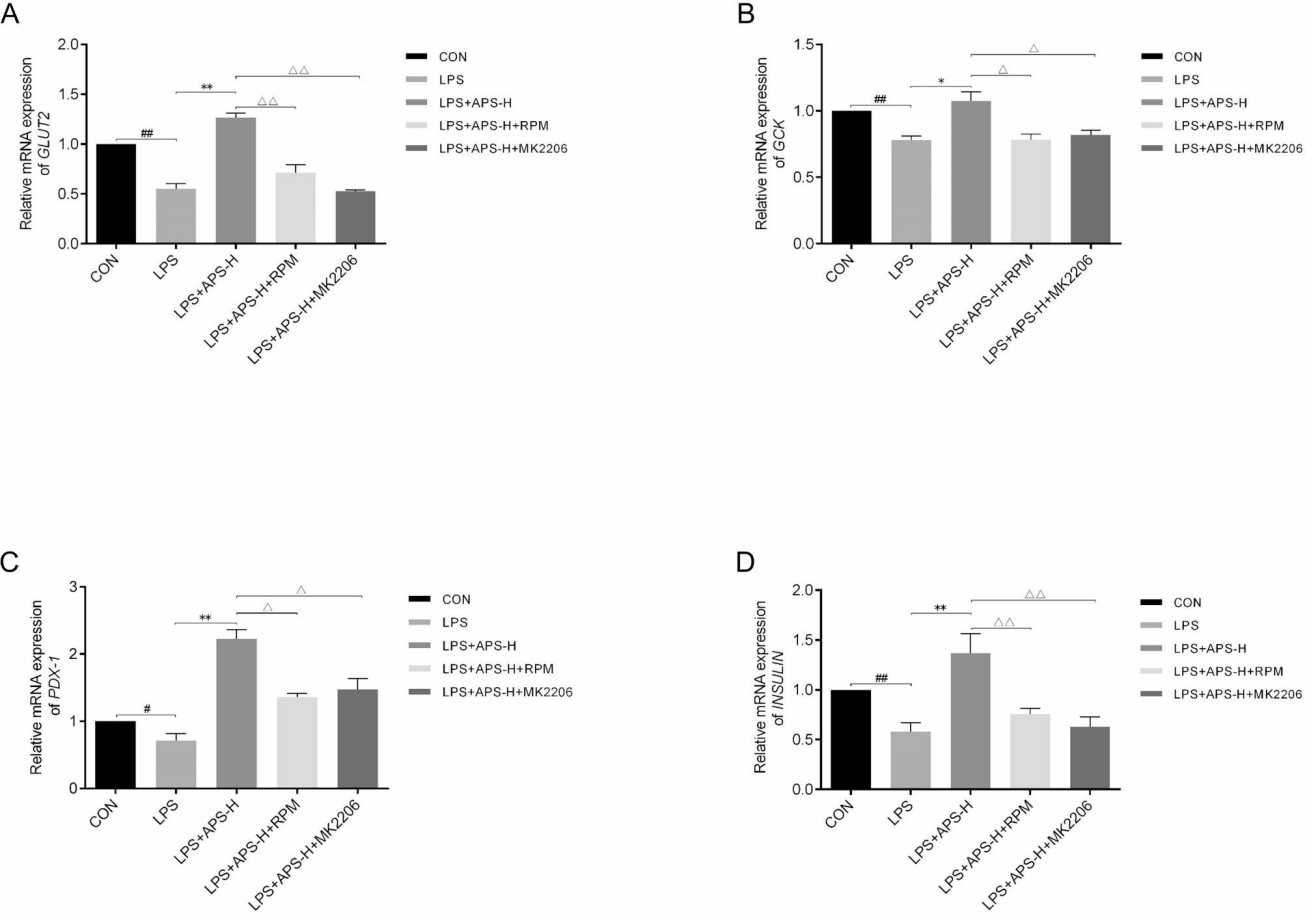
Blockade of the Akt/mTOR pathway downregulated *GLUT2*, *GCK*, *PDX-1*, and *INS* upregulated by APS in LPS-stimulated INS-1 cells. INS-1 cells were treated as indicated for 24 h. Untreated cells were included as a control. The mRNA levels of *GLUT2* **(A)**, *GCK* **(B)**, *PDX-1* **(C)**, and *INS* **(D)** were determined by qRT-PCR. *n* = 3, *^,#,Δ^*P* < 0.05, **^,##,ΔΔ^*P* < 0.01

**Fig. 8:**
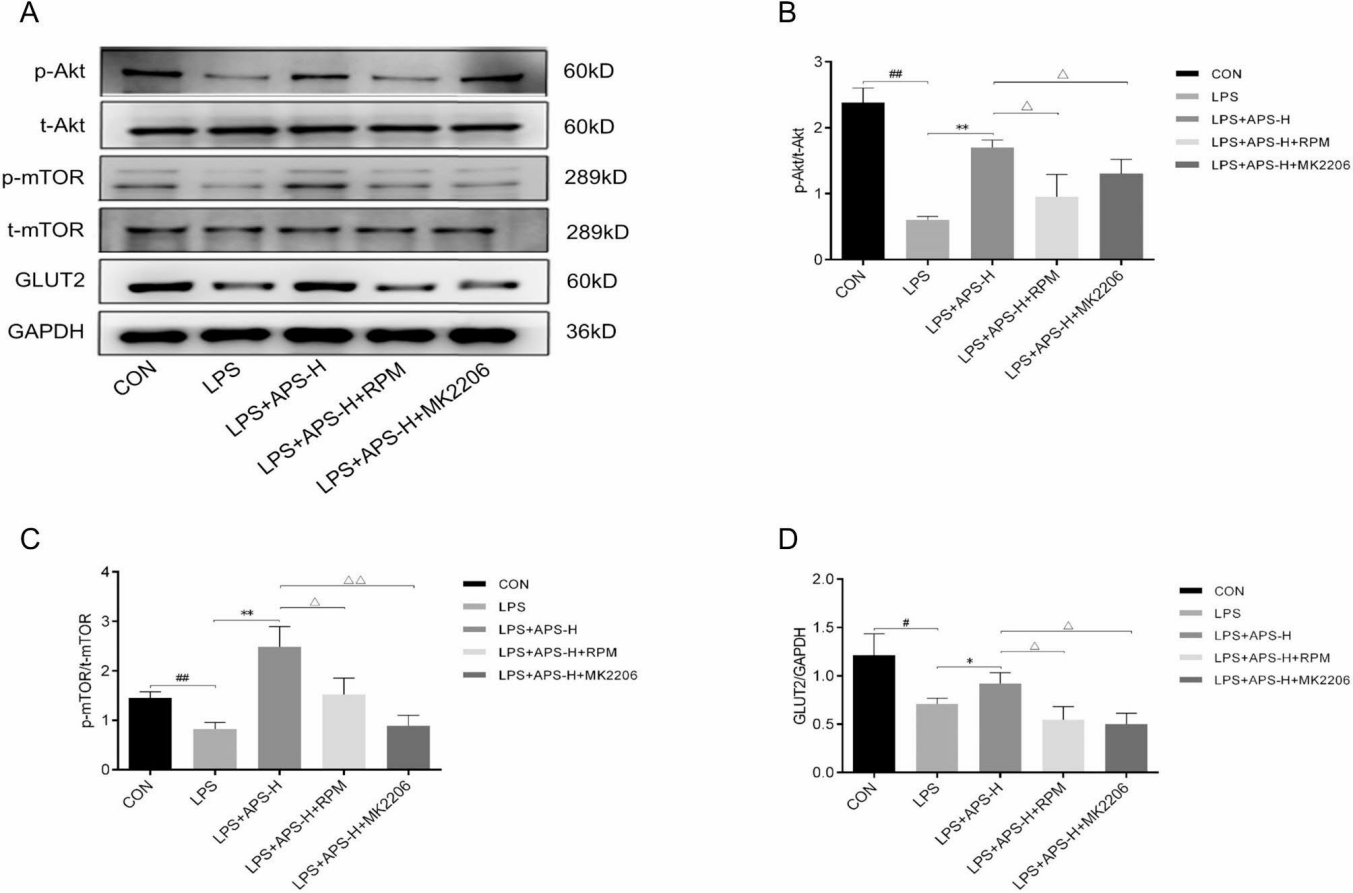
MK2206 and RPM inhibited the Akt/mTOR pathway. INS-1 cells were treated as indicated for 24 h. Untreated cells were included as a control. The levels of the indicated proteins were determined by western blot analysis. **(A)** Representative gel images. **(B)** Quantified protein ratios of p-Akt/t-Akt. **(C)** Quantified protein ratios of p-mTOR/t-mTOR. **(D)** Quantified GLUT2 protein expression. *n* = 3, *^,#,Δ^*P* < 0.05, **^,##,ΔΔ^*P* < 0.01

To verify that the Akt/mTOR pathway also mediates the upregulation of GLUT2, GCK, PDX1, and INS by APS in LPS-stimulated INS-1 cells, the effects of MK2206 and RPM on the expression of these genes were tested. The qRT-PCR results revealed that treatment with 1 µM MK2206 or 5 µM RPM for 24 h abolished the upregulation of *GLUT2*, *GCK*, *PDX-1*, and *INS* by APS (*P* < 0.05, Fig. 7A–D). In particular, the effects of MK2206 or RPM on GLUT2 were confirmed by western blot analysis (*P* < 0.05, Fig. 8A–D). Together, these data supported that APS restored GLUT2, GCK, PDX-1, and INS expression downregulated by LPS via Akt/mTOR-dependent mechanisms. Finally, the western blot data confirmed the inhibition of the Akt/mTOR pathway by MK2206 or RPM (Fig. 8B–C).

## Discussion

Although gut microbiota-derived LPS can activate proinflammatory pathways leading to β-cell dysfunction and decreased insulin secretion [9, 10], the molecular mechanisms involved are largely undefined. In this study, LPS reduced GSIS of INS-1 cells by downregulating GSIS-related genes including *GLUT2*, *GCK*, *PDX-1*, and *INS*. Treatment with APS upregulated these GSIS-related genes and restored GSIS impaired by LPS. The mechanistic studies revealed that the Akt/mTOR pathway functions as a mediator of the restorative effects of APS. These findings suggest the APS may hold promise as a potential therapy for T2DM by mitigating β-cell dysfunction and insulin secretion impairment caused by microbiota-derived inflammatory stimulators such as LPS.

Inflammatory factors such as TNF-α and IL-1β cause β-cell injury and dysfunction in diabetes [26]. In addition, LPS has been shown to induce oxidative stress and increase IL-1β expression in INS-1 cells [27]. LPS also has been shown to trigger cell apoptosis and reduce GSIS in MIN6 cells [28]. Notably, LPS stimulation of β-cells has been used as an *in-vitro* model of inflammatory pancreatic islet injury [27, 29]. In the present study, LPS was found to upregulate TNF-α and IL-1β in INS-1 cells, indicating activation of multiple inflammatory pathways. Moreover, GSIS from INS-1 cells was diminished by LPS stimulation. These results were mostly in line with previous reports. However, LPS did not induce cell apoptosis in the current study, which could be attributed to the activation of cytoprotective autophagy [27].

In China, herbal medicines have been used to treat T2DM for centuries [30, 31]. APS is extracted from the stems or dried roots of *A. membranaceus* (known as Huangqi in China) and is a popular Chinese herbal medicine. APS has been shown to alleviate INS resistance and T2DM in animal models through mechanisms involving the liver and muscle [32, 33]. Nevertheless, how APS affects pancreatic β-cells is not clear. In the current study, APS restored GSIS in LPS-stimulated INS-1 cells by upregulating GLUT2, GCK, PDX-1, and INS. GLUT2 is the predominant glucose transporter in pancreatic β-cells and is a master regulator of GSIS [23, 24]. Single-nucleotide polymorphisms in *GLUT2* have been shown to predict the transition from impaired glucose tolerance to T2DM in participants of the Finnish Diabetes Prevention Study [34]. GCK is mainly expressed in the liver and pancreas. In pancreatic β-cells, GCK acts as a glucose-sensing molecule and is a principal gatekeeper of GSIS [35]. Meanwhile, PDX-1 is a transcription factor that controls β-cell production, differentiation, and function as well as glucose-dependent expression of the *INS* gene [36]. The upregulation of GLUT2, GCK, and PDX-1 by APS provided strong mechanistic support for its restorative effects on GSIS impaired by LPS.

Genetic studies have shown that the kinase activity of Akt is critical for maintaining β-cell homeostasis and INS secretion [37]. A key effector of Akt is mTOR. A recent study has demonstrated that 17β-estradiol (estrogen 2) promotes INS secretion via the Akt/mTOR/GLUT2 pathway [25]. In the current study, LPS downregulated p-Akt and p-mTOR, which were restored by APS. More importantly, blocking the Akt/mTOR pathway with MK2206 or RPM abolished the restorative effects of APS on GSIS as well as on the expression of GSIS-related genes impaired by LPS. These results support that the Akt/mTOR/GLUT2 pathway is a critical mediator of the restorative effects of APS on GSIS.

APS has been reported to regulate immune functions by promoting immune cell proliferation and stimulating cytokine release [38]. In the present study, APS upregulated TNF-α and IL-1β (Sup. Figure 2). These cytokine signaling pathways can crosstalk with the Akt/mTOR pathway to influence GSIS. However, further investigation is needed to fully understand the mechanisms underlying the pharmacological action of APS in pancreatic β-cells.

## Conclusions

APS treatment restored GSIS of INS-1 cells impaired by LPS through the Akt/mTOR/GLUT2 pathway. APS may hold promise as a novel T2DM therapy by mitigating pancreatic β-cell dysfunction caused by metabolic endotoxemia.

### Electronic supplementary material

Below is the link to the electronic supplementary material.

Supplementary Material 1: Supplementary Figures 1 and 2.

## Data Availability

The datasets generated and/or analyzed during the current study are available in the [BioProject] repository [http://www.ncbi.nlm.nih.gov/bioproject/956873; ID: PRJNA956873].

## List of Abbreviations

LPS: Lipopolysaccharide
APS: Astragalus polysaccharide
INS: Insulin
GSIS: Glucose-stimulated insulin secretion
KEGG: Kyoto Encyclopedia of Genes and Genomes
GLUT2: Glucose transporter 2
GCK: Glucokinase
PDX-1: Pancreatic duodenal homeobox-1
T2DM: Type 2 diabetes mellitus
Akt: Protein kinase B
mTOR: Mammalian target of rapamycin
RPM: Rapamycin
CCK-8: Cell counting kit-8

## References

[1] Tinajero MG, Malik VS (2021). An update on the epidemiology of type 2 diabetes: A Global Perspective. Endocrinol Metab Clin North Am.

[2] Seino S, Sugawara K, Yokoi N, Takahashi H (2017). β-Cell signalling and insulin secretagogues: a path for improved diabetes therapy. Diabetes Obes Metab.

[3] Lv W, Wang X, Xu Q, Lu W (2020). Mechanisms and characteristics of Sulfonylureas and Glinides. Curr Top Med Chem.

[4] Filippatos TD, Panagiotopoulou TV, Elisaf MS (2014). Adverse Effects of GLP-1 receptor agonists. Rev Diabet Stud.

[5] Jo S, Fang S (2021). Therapeutic strategies for diabetes: Immune Modulation in pancreatic β cells. Front Endocrinol (Lausanne).

[6] Musso G, Gambino R, Cassader M (2011). Interactions between gut microbiota and host metabolism predisposing to obesity and diabetes. Annu Rev Med.

[7] Gomes JMG, Costa JA, Alfenas RdCG (2017). Metabolic endotoxemia and diabetes mellitus: a systematic review. Metabolism.

[8] Huang X, Yan D, Xu M, Li F, Ren M, Zhang J (2019). Interactive association of lipopolysaccharide and free fatty acid with the prevalence of type 2 diabetes: a community-based cross-sectional study. J Diabetes Investig.

[9] Garay-Malpartida HM, Mourão RF, Mantovani M, Santos IA, Sogayar MC, Goldberg AC (2011). Toll-like receptor 4 (TLR4) expression in human and murine pancreatic beta-cells affects cell viability and insulin homeostasis. BMC Immunol.

[10] Hsieh PS, Chan JY, Shyu JF, Chen YT, Loh CH (2008). Mild portal endotoxaemia induces subacute hepatic inflammation and pancreatic beta-cell dysfunction in rats. Eur J Clin Invest.

[11] Sun S, Yang S, An N, Wang G, Xu Q, Liu J (2019). Astragalus polysaccharides inhibits cardiomyocyte apoptosis during diabetic cardiomyopathy via the endoplasmic reticulum stress pathway. J Ethnopharmacol.

[12] Yan X, Lu Q, Zeng L, Li X, Liu Y, Du X (2020). Synergistic protection of astragalus polysaccharides and matrine against ulcerative colitis and associated lung injury in rats. World J Gastroenterol.

[13] Li W, Hu X, Wang S, Jiao Z, Sun T, Liu T (2020). Characterization and anti-tumor bioactivity of astragalus polysaccharides by immunomodulation. Int J Biol Macromol.

[14] Luo MJ, Wang Y, Chen SY, Yang ZM (2022). Astragalus Polysaccharides alleviate type 2 Diabetic rats by reversing the Expressions of Sweet taste receptors and genes related to Glycolipid Metabolism in Liver. Front Pharmacol.

[15] Gu C, Zeng Y, Tang Z, Wang C, He Y, Feng X (2015). Astragalus polysaccharides affect insulin resistance by regulating the hepatic SIRT1-PGC-1α/PPARα-FGF21 signaling pathway in male Sprague Dawley rats undergoing catch-up growth. Mol Med Rep.

[16] Deng S, Yang L, Ma K, Bian W (2021). Astragalus polysaccharide improve the proliferation and insulin secretion of mouse pancreatic beta cells induced by high glucose and palmitic acid partially through promoting mir-136-5p and mir-149-5p expression. Bioengineered.

[17] Ge QM, Du SC, Bian F, Lin N, Su Q. Effects of lipopolysaccharides on TLR4 expression in INS-1 rat insulinoma cells. Cell Mol Biol (Noisy-le-grand). 2011;57 Suppl:Ol1513-9. 21699766

[18] Liu J, Chen S, Ren W, Liu J, Yang P, Chen Z (2017). Lipopolysaccharide-induced suppression of periodontal ligament cell proliferation and apoptosis are strengthened under high glucose conditions. Arch Oral Biol.

[19] Kanehisa M, Furumichi M, Sato Y, Kawashima M, Ishiguro-Watanabe M (2023). KEGG for taxonomy-based analysis of pathways and genomes. Nucleic Acids Res.

[20] Zhou Z, Meng M, Ni H (2017). Chemosensitizing effect of Astragalus Polysaccharides on nasopharyngeal carcinoma cells by inducing apoptosis and modulating expression of Bax/Bcl-2 ratio and caspases. Med Sci Monit.

[21] Corkey BE, Deeney JT, Merrins MJ (2021). What regulates basal insulin secretion and causes hyperinsulinemia?. Diabetes.

[22] Henquin JC (2009). Regulation of insulin secretion: a matter of phase control and amplitude modulation. Diabetologia.

[23] Guillam MT, Dupraz P, Thorens B (2000). Glucose uptake, utilization, and signaling in GLUT2-null islets. Diabetes.

[24] Low BSJ, Lim CS, Ding SSL, Tan YS, Ng NHJ, Krishnan VG (2021). Decreased GLUT2 and glucose uptake contribute to insulin secretion defects in MODY3/HNF1A hiPSC-derived mutant beta cells. Nat Commun.

[25] Bian C, Bai B, Gao Q, Li S, Zhao Y (2019). 17β-Estradiol regulates glucose metabolism and insulin secretion in rat islet β cells through GPER and Akt/mTOR/GLUT2 pathway. Front Endocrinol (Lausanne).

[26] Eizirik DL, Sammeth M, Bouckenooghe T, Bottu G, Sisino G, Igoillo-Esteve M (2012). The human pancreatic islet transcriptome: expression of candidate genes for type 1 diabetes and the impact of pro-inflammatory cytokines. PLoS Genet.

[27] Zhu LB, Cao MM, Wang J, Su Y, Jiang W, Liu GD (2019). Role of autophagy in LPSinduced inflammation in INS1 cells. Mol Med Rep.

[28] Chen D, Cao D, Sui P (2019). Tetramethylpyrazine relieves LPS-induced pancreatic β-cell Min6 injury via regulation of miR-101/MKP-1. Artif Cells Nanomed Biotechnol.

[29] Xu YN, Wang Z, Zhang SK, Xu JR, Pan ZX, Wei X (2022). Low-grade elevation of palmitate and lipopolysaccharide synergistically induced beta-cell damage via inhibition of neutral ceramidase. Mol Cell Endocrinol.

[30] Meng X, Liu X, Tan J, Sheng Q, Zhang D, Li B (2023). From Xiaoke to diabetes mellitus: a review of the research progress in traditional chinese medicine for diabetes mellitus treatment. Chin Med.

[31] Su M, Hu R, Tang T, Tang W, Huang C (2022). Review of the correlation between chinese medicine and intestinal microbiota on the efficacy of diabetes mellitus. Front Endocrinol (Lausanne).

[32] Wei Z, Weng S, Wang L, Mao Z (2018). Mechanism of Astragalus polysaccharides in attenuating insulin resistance in rats with type 2 diabetes mellitus via the regulation of liver microRNA–203a–3p. Mol Med Rep.

[33] Zou F, Mao X-q, Wang N, Liu J, Ou-Yang J-p (2009). Astragalus polysaccharides alleviates glucose toxicity and restores glucose homeostasis in diabetic states via activation of AMPK. Acta Pharmacol Sin.

[34] Laukkanen O, Lindstrom J, Eriksson J, Valle TT, Hamalainen H, Ilanne-Parikka P (2005). Polymorphisms in the SLC2A2 (GLUT2) gene are associated with the conversion from impaired glucose tolerance to type 2 diabetes: the finnish diabetes Prevention Study. Diabetes.

[35] Bell GI, Pilkis SJ, Weber IT, Polonsky KS (1996). Glucokinase mutations, insulin secretion, and diabetes mellitus. Annu Rev Physiol.

[36] McKinnon CM, Docherty K (2001). Pancreatic duodenal homeobox-1, PDX-1, a major regulator of beta cell identity and function. Diabetologia.

[37] Bernal-Mizrachi E, Szabolcs Fatrai JD, Johnson M, Ohsugi K, Otani Z, Han (2004). Defective insulin secretion and increased susceptibility to experimental diabetes are induced by reduced akt activity in pancreatic islet beta cells. J Clin Invest.

[38] Zheng Y, Ren W, Zhang L, Zhang Y, Liu D, Liu Y (2020). A review of the pharmacological action of Astragalus Polysaccharide. Front Pharmacol.

