# Correction: Astragalus polysaccharide restores insulin secretion impaired by lipopolysaccharides through the protein kinase B/mammalian target of rapamycin/glucose transporter 2 pathway

**DOI:** 10.1186/s12906-023-04260-w

**Published:** 2023-11-20

**Authors:** Xiaodan Ren, Ying Dai, Mengya Shan, Jing Zheng, Zhongyi Zhang, Tao Shen

**Affiliations:** 1 School of Basic Medicine, Chengdu University of Traditional Chinese Medicine, No. 37, Shi-Er-Qiao Road, Jinniu District, 610075, 610075, Chengdu, , Chengdu, Sichuan China; 2 Department of Integrative Medicine, Xinqiao Hospital, Army Medical University, Chongqing, China

**Correction: BMC Complement Med Ther 23, 358 (2023)**

**https://doi.org/10.1186/s12906-023-04188-1**

Following publication of the original article [1], the authors identified two errors that need to be corrected.

Error Description:

In Additional file 1, Supplemental Figure 1 was incorrectly presented as Fig. 5A western blot experimental pictures.

In Additional file 1, Supplemental Figure 2 was incorrectly presented as Fig. 8A western blot experimental pictures.

Correction:

The correct Supplemental Figure 1 should be “**Neither LPS nor APS had significant effects on INS-1 cell apoptosis**”.

The correct Supplemental Figure 2 should be “**The effects of LPS and APS on inflammatory factors**”.

These corrections do not impact the overall findings and conclusions of the paper. They would like to assure readers that the corrected supplementary material does not alter the interpretations or validity of the research.

The original article has been corrected.

### Supplementary Information

**Additional file 1: Supplemental Figure 1. **Neither LPS nor APS had significant effects on INS-1 cell apoptosis. INS-1 cells were treated as indicated for 24 h. Cell apoptosis was evaluated with flow cytometry as described in materials and methods. **Supplemental Figure 2. **The effects of LPS and APS on inflammatory factors. INS-1 cells were treated as indicated for 24 h. The mRNA levels of indicated inflammatory factors were determined with qRT-PCR. *n*=3, ^#^*P*<0.05, ^##^*P*<0.01.
