# Supplementary material for: Astragalus polysaccharide restores insulin secretion impaired by lipopolysaccharides through the protein kinase B /mammalian target of rapamycin/glucose transporter 2 pathway
Source: BMC Complement Med Ther. 2023 Oct 10;23:358. doi: 10.1186/s12906-023-04188-1 (PMC10563267; doi:10.1186/s12906-023-04188-1)
Supplement: Supplementary file 1 — Supplementary Material 1: Supplementary Figures 1 and 2. [file 12906_2023_4188_MOESM1_ESM.pdf]

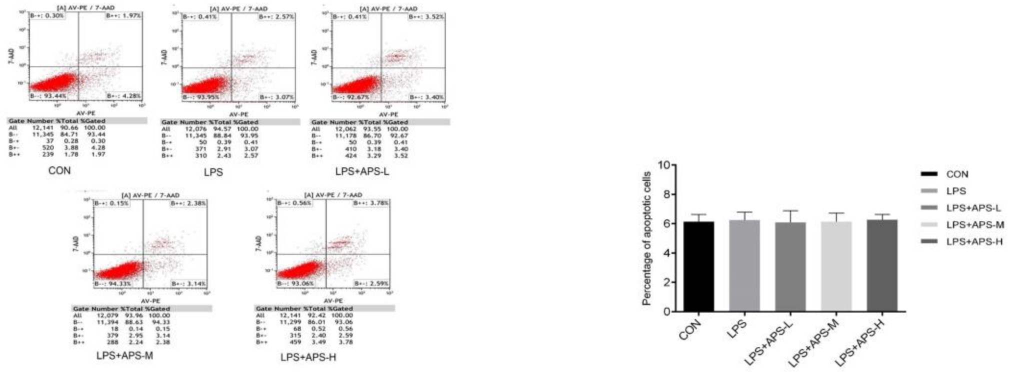

Supplemental Figure 1. Neither LPS nor APS had significant effects on INS-1 cell apoptosis. INS-1 cells were treated as indicated for 24 h. Cell apoptosis was evaluated with flow cytometry as described in materials and methods.

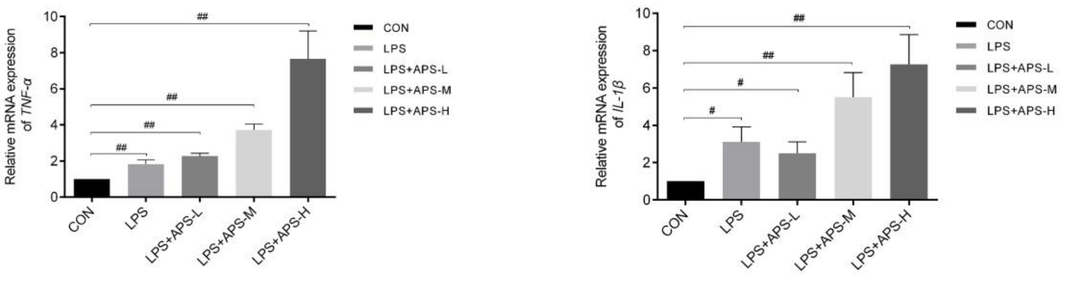

Supplemental Figure 2. The effects of LPS and APS on inflammatory factors. INS-1 cells were treated as indicated for 24 h. The mRNA levels of indicated inflammatory factors were determined with qRT-PCR. *n*=3, $^{\#}P<0.05$, $^{\#\#}P<0.01$.
